# Time Course of Autonomic Symptoms in Postural Orthostatic Tachycardia Syndrome (POTS) Patients: Two-Year Follow-Up Results

**DOI:** 10.3390/ijerph17165872

**Published:** 2020-08-13

**Authors:** Franca Dipaola, Caterina Barberi, Elena Castelnuovo, Maura Minonzio, Roberto Fornerone, Dana Shiffer, Beatrice Cairo, Antonio Roberto Zamuner, Franca Barbic, Raffaello Furlan

**Affiliations:** 1 Internal Medicine, Humanitas Clinical and Research Center—IRCCS, Rozzano, 20089 Milan, Italy; franca.dipaola@humanitas.it (F.D.); caterina.barberi@humanitas.it (C.B.); elena.castelnuovo@humanitas.it (E.C.); maura.minonzio@humanitas.it (M.M.); robertofornerone@tiscali.it (R.F.); dana.shiffer@humanitas.it (D.S.); franca.barbic@humanitas.it (F.B.); 2 Department of Biomedical Sciences, Humanitas University, Pieve Emanuele, 20090 Milan, Italy; 3 Department of Biomedical Sciences for Health, University of Milan, 20122 Milan, Italy; beatrice.cairo@unimi.it; 4 Departamento de Kinesiología, Universidad Católica del Maule, 3605 Talca, Chile; beto.zam@gmail.com

**Keywords:** POTS, syncope, orthostatic hypotension, working

## Abstract

Postural orthostatic tachycardia syndrome (POTS) is a multifactorial condition capable of chronically reducing the quality of life and the work ability of patients. The study aim was to assess the burden of autonomic symptoms in a cohort of POTS patients over 2 years. Patients’ clinical profiles were assessed by the 31-item Composite Autonomic Symptom Score questionnaire (COMPASS 31) and a visual analog scale (VAS). One-way ANOVA for repeated measures followed by Dunnett’s post-hoc test were used to compare symptoms at baseline and at 1 and 2 years. Out of 42 enrolled patients, 25 had a 1-year follow-up and 12 had a 2-year follow-up. At baseline, the reported burden of autonomic symptoms was high (overall COMPASS 31 = 49.9 ± 14.3 /100). Main complaints were related to orthostatic intolerance according to both COMPASS 31 and VAS. Fourteen patients were rendered inactive because of symptoms. At 1-year follow-up, a statistically significant improvement in pupillomotor function and overall score was detected by the COMPASS 31. These findings were confirmed at 2 years, together with a significant reduction in quality of life impairment, assessed by VAS. However, these improvements did not change patients’ occupational status. Awareness of POTS diagnosis, patient monitoring, and tailored therapies can help to improve patients’ condition.

## 1. Introduction

Postural orthostatic tachycardia syndrome (POTS), also named postural tachycardia syndrome, is a multifactorial cardiovascular autonomic disorder characterized by all of the following [1]:

– an increase in heart rate of ≥ 30 bpm, or ≥ 40 bpm for those under age 19, within 10 minutes of standing from a supine position;
– sustained tachycardia (> 30 s);
– absence of orthostatic hypotension (a fall in blood pressure of ≥ 20/10 mm Hg);
– symptoms characterized by frequent occurrence and chronic duration (≥ 6 months).

Orthostatic tachycardia may be accompanied by symptoms of cerebral hypoperfusion (i.e., light-headedness, blurred vision, cognitive difficulties, generalized weakness) and sympathetic hyperactivity (i.e., palpitations, chest pain, tremors) that are relieved by assuming the supine position [2]. Patients with POTS faint occasionally. However, pre-syncope is much more common [1,2]. 

In addition, many patients experience other chronic conditions whose symptoms cannot be mechanistically explained by postural intolerance or excessive tachycardia [2]. These include the following:

– visceral pain and dysmotility of the upper or lower gastrointestinal tract, bladder, and other organs [3,4];
– chronic fatigue [5,6], fibromyalgia [7], and sleep disturbances [8];
– joint hypermobility and Ehlers–Danlos syndrome type III [9,10];
– chronic headache, including migraine and orthostatic headache [11,12].

In particular, gastrointestinal disturbances (i.e., abdominal pain, heartburn, irregular bowel movements, diarrhea, or constipation) are common in POTS, tend to be prolonged (hours), and occur multiple times a week. They generally do not improve in the supine position [3]. POTS-associated symptoms may develop insidiously, but patients often report the onset after an acute stressor such as pregnancy, major surgery, or a presumed viral illness [13].

The pathophysiology of POTS is poorly understood [1,2]. Proposed mechanisms include neuropathic and hyperadrenergic dysregulation, volume depletion, physical deconditioning, and autoimmunity [14,15,16,17,18]. 

The prevalence of POTS is estimated to be between 0.2% and 1.0%, affecting up to 3 million people in the United States. Most cases arise between ages 13 and 50, with a female-to-male ratio of around 5:1 [19]. The exact prevalence in Europe is unknown [20,21]. Patients are often underdiagnosed or misdiagnosed and are evaluated on average by seven doctors before receiving a POTS diagnosis. The median diagnostic delay is 24 months, with peaks reaching 10 years [22]. The chronic and usually systemic symptoms, anxiety, and the frustration caused by the difficulty in obtaining medical help can significantly lower the patients’ quality of life. As a result of orthostatic intolerance, patients can progressively become physically deconditioned [23,24,25], thus reducing their working capacity. In some cases, they may finally become wheelchair users or bed-bound and are often unable to continue in education or employment (25%) [20,26]. Life expectancy is thought to be unaffected, but disability is considerable and equivalent to that found in congestive heart failure and chronic obstructive pulmonary disease [1,20]. 

The burden of symptoms and quality of life of POTS patients were previously described in cross-sectional studies [27,28,29,30,31], but prospective observations are lacking. 

The aim of the present study is to quantitatively and semi-quantitatively assess the burden of autonomic symptoms in a cohort of POTS patients followed over a period of 2 years.

## 2. Methods

All POTS patients evaluated regularly in our clinic between August 2016 and December 2018 were enrolled in the study. POTS diagnosis was based on current consensus criteria [1,21].

All patients were examined every four months for a total follow-up time of 2 years. During the visits, a 75° head-up tilt test was performed [32] and the patient’s clinical profile was assessed by administering the 31-item Composite Autonomic Symptom Score questionnaire (COMPASS 31) [33] and a visual analog scale (VAS). At the end of each evaluation, the physicians proposed optimization of the ongoing therapy, if necessary, according to their clinical judgment. All patients were strongly recommended to engage in a regular physical exercise program aimed at reconditioning and strengthening the muscles of the lower limbs.

The COMPASS 31 [33] is a self-assessment tool used to evaluate autonomic symptoms. It consists of 31 items which aim to evaluate six domains of autonomic functions (orthostatic intolerance, 4 items; vasomotor, 3 items; secretomotor, 4 items; gastrointestinal, 12 items; bladder, 3 items; pupillomotor, 5 items). The overall score is achieved by calculating the raw domain scores, which are determined by adding up the points of the questions in each domain. The raw score of each domain is multiplied by a weight index which yields the final domain score. The sum of all domain scores produces the total score, which ranges from 0 (normal) to 100 (the worst condition) [33].

In addition, patients were asked to fill out a modified VAS applied to 13 predefined major symptoms related to five impaired autonomic functions. Symptoms are as follows: 1. orthostatic intolerance: impaired quality of life, upright dizziness, interscapular pain during exercise; 2. excretory function: reduced sweating, reduced lacrimation, reduced salivation; 3. genito-urinary function: urinary retention, urinary incontinence, reduced sexual desire, reduced sexual activity; 4. gastrointestinal function: dysphagia, constipation; 5. visual acuity. For each symptom, the stem question was as follows: “How severe is your symptom today?” The patient had to place a mark on a 10 cm long line, in which 0 corresponded to the absence of a symptom and 10 to the symptom’s maximum intensity. The final value for each symptom was obtained by measuring the distance in cm from 0 to the mark placed by the patient. This method, although validated and used mostly for pain intensity quantification [34], proved to be rapid and outright in our clinical experience [35]. It allowed for a reliable semi-quantitative evaluation of the changes in symptom intensity over time.

We arbitrarily chose to compare symptoms at baseline, 1 year, and 2 years to describe any changes at more clinically significant time intervals.

The study was approved by the local ethics committee (protocol no. 150/16, authorization no. 1611) and all patients provided informed consent to participate.

Descriptive data are presented as mean (±SD) for continuous variables and as numbers and percentages for categorical variables. Differences between patients at baseline and at 1-year and 2-year follow-up were evaluated using the chi-square test and the Fischer exact test whenever appropriate. One-way ANOVA for repeated measures followed by Dunnett’s post-hoc test were used to evaluate changes in autonomic symptom assessment over time. *p* values <0.05 were considered significant. Statistical analyses were conducted using GraphPad Prism^TM^ Software (Version 8.0, GraphPad Software, 2365 Northside Dr.Suite 560San Diego, CA, USA).

## 3. Results

Forty-two patients (females, n = 36) were enrolled in the study. One-year follow-up was achieved in 25 patients, while 12 patients completed the two-year follow-up. Patients lost to follow-up dropped out of the study for personal or organizational reasons. None of the patients died at two years of follow-up or reported a resolution of the clinical picture.

POTS was diagnosed at time of enrolment in 21 out of 42 patients. The overall average time from diagnosis to enrolment was 6.4 ± 17 months.

The demographic and clinical characteristics of POTS patients at baseline and at 1- and at 2- year follow-up are summarized in Table 1. No statistically significant differences were observed between patients at baseline or at 1-year and 2-year follow-up. Thirty one out of 42 patients were on drug therapy at baseline. The drugs used are summarized in Table 1. Ivabradine and beta blockers were the two most commonly prescribed drugs at each follow-up time. Twenty-one patients were on monotherapy while 10 were taking two or more drugs. 

At 1-year follow-up, 19 out of 25 patients were taking medications. After medical evaluation, 15 patients changed the pharmacological class or increased the dosage of the ongoing drugs, two individuals reduced the dosage, and in two other people, the therapy remained unchanged. At 2-year follow-up, eight out of 12 patients were taking at least one drug; three patients increased the dosage of the drugs in comparison to the 1-year follow-up, two reduced it, and for the remaining three patients, the therapy was unmodified.

Work activity status was known for 36 patients out of 42. Twenty-two patients were active workers or students. The remaining 14 had stopped working or studying because of POTS-related symptoms. The most represented categories were employees and students, both among active and inactive patients. None of the patients changed their occupational status during the follow-up period.

**Figure 1:**
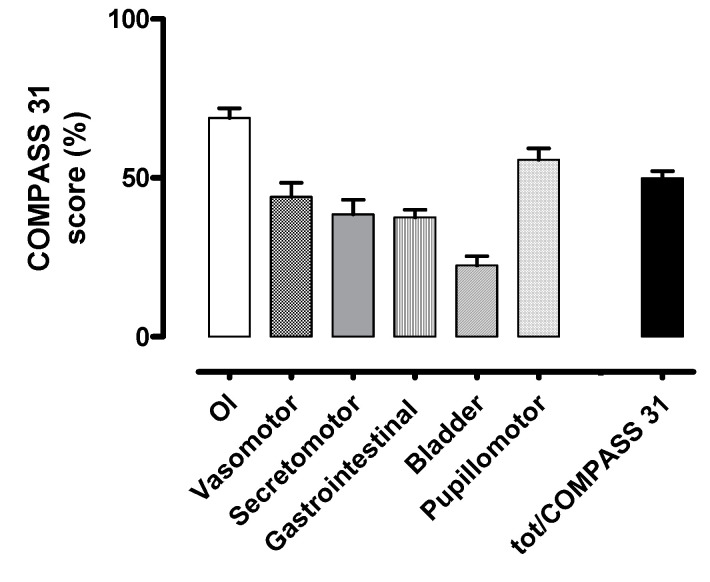
Baseline autonomic symptom assessment by COMPASS 31 questionnaire. OI indicates orthostatic intolerance domain; Vasomotor, vasomotor function domain; Secretomotor, secretomotor function domain; Gastrointestinal, gastrointestinal function domain; Bladder, bladder function domain; Pupillomotor, pupillomotor function domain; tot/COMPASS 31, total COMPASS 31 score. The scores are here represented as a percentage of the maximum value obtainable in each domain.

The burden of autonomic symptoms assessed at baseline by the COMPASS 31 is shown in Figure 1. 

COMPASS 31 domains’ weighted scores and total score are reported in Table 2.

According to Sletten et al. [33], the maximum raw score for each domain was determined and each domain was assigned a weight factor based on the authors’ current perception of the importance of domains for reflecting autonomic failure, so that the minimal weighted score for the instrument equals 0 and the maximum weighted score equals 100.

**Figure 2:**
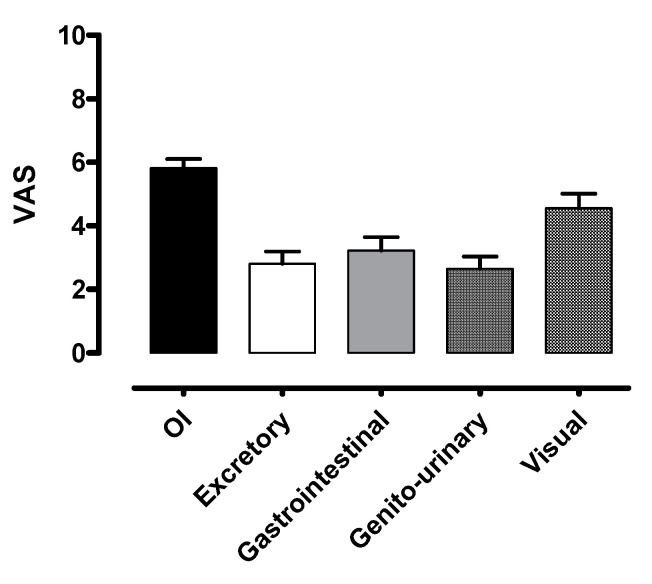
Autonomic symptom assessment at baseline by modified VAS. OI indicates orthostatic intolerance; Excretory, excretory function; Gastrointestinal, gastrointestinal function; Genito-urinary, genito-urinary function; Visual, visual acuity.

The autonomic symptoms as represented by VAS at baseline are described in Figure 2.

For each function, the score can range from “0” (absence of symptoms) to “10” (maximum intensity of symptoms).

The main symptoms described were related to orthostatic intolerance and gastrointestinal function according to the COMPASS 31 (weighted score 27.52/40 and 9.37/25, respectively) and to orthostatic intolerance and visual acuity according to the VAS (score 5.82/10 and 4.56/10, respectively).

**Figure 3:**
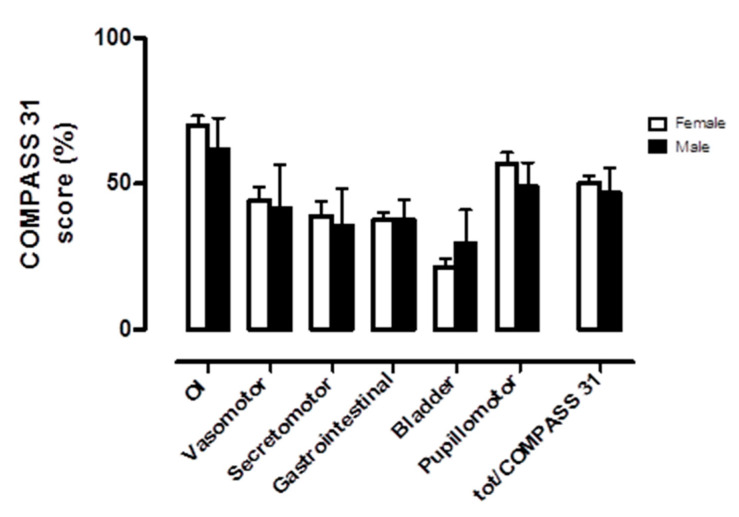
Baseline autonomic symptom assessment by COMPASS 31 according to gender. OI indicates orthostatic intolerance domain; Vasomotor, vasomotor function domain; Secretomotor, secretomotor function domain; Gastrointestinal, gastrointestinal function domain; Bladder, bladder function domain; Pupillomotor, pupillomotor function domain; tot/COMPASS 31, total COMPASS 31 score. The scores are here represented as a percentage of the maximum value obtainable in each domain.

**Figure 4:**
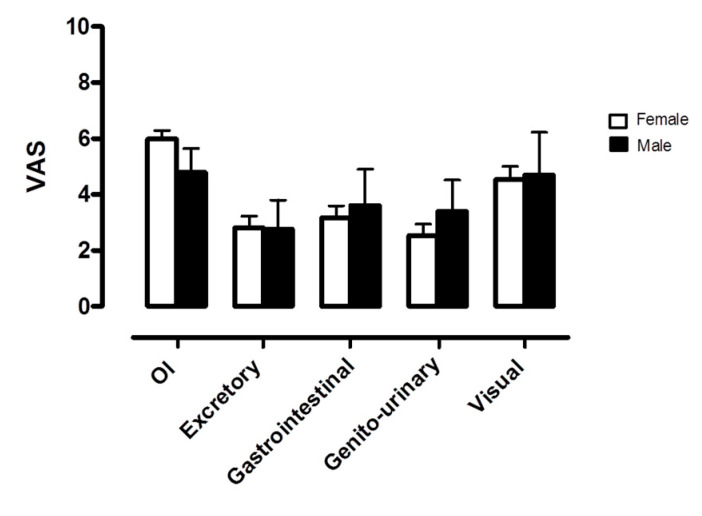
Autonomic symptom assessment at baseline by modified VAS according to gender. OI indicates orthostatic intolerance; Excretory, excretory function; Gastrointestinal, gastrointestinal function; Genito-urinary, genito-urinary function; Visual, visual acuity.

Patients were analyzed at baseline according to gender. There were no statistically significant differences in demographic and clinical characteristics (see Table 3). Orthostatic intolerance was the most represented domain in both genders, as shown in Figure 3 and Figure 4. No significant differences were observed in self-assessed autonomic symptoms at baseline when comparing females and males.

COMPASS 31 domains’ weighted scores and total score according to gender are reported in Table 4.

The trend over time of the autonomic symptoms, as assessed by COMPASS 31 and VAS, is summarized in Table 5 and Table 6, respectively. By the use of COMPASS 31, the pupillomotor function domain and the overall score were significantly reduced at 1-year follow-up.

Domain weighted scores and total score are calculated according to Sletten et al. [33].

VAS detected no significant difference over time in any of the domains assessed (see Table 6 below).

**Figure 5:**
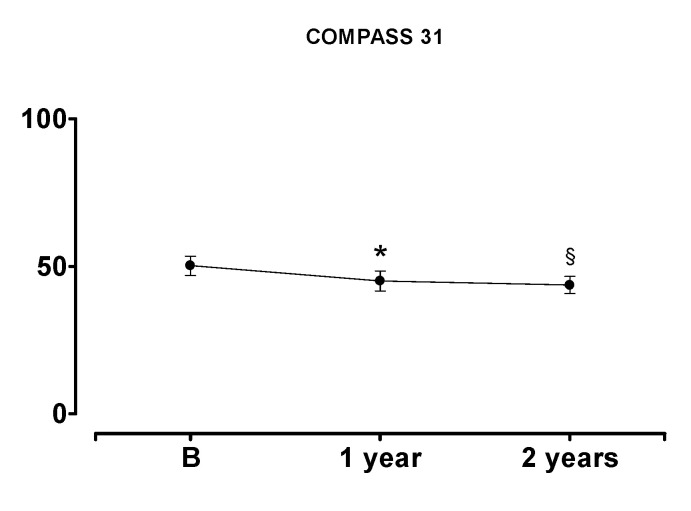
COMPASS 31 total score comparison between baseline and 1-year and 2-year follow-up. B indicates baseline. * indicates *p* < 0.05 12 months vs. baseline; ^§^ indicates *p* < 0.05 24 months vs. baseline. Results are expressed as mean ± SD.

In the 12 patients who completed the 2-year follow-up, the overall COMPASS 31 was significantly reduced compared to baseline at both 1-year and 2-year follow-up, thus confirming a progressive global clinical improvement (see Figure 5). A significant improvement in pupillomotor function at the 2-year follow-up was also observed (see Table 7 below).

Domain weighted scores and total score are calculated according to Sletten et al. [33].

VAS showed no significant changes over time for any of the symptoms explored, except for a reduction in quality of life impairment at 2 years compared to baseline (see Table 8 below). 

## 4. Discussion

Our results indicate that POTS patients are characterized by a high burden of autonomic symptoms, as confirmed by the COMPASS 31 average total score of 49.9, out of a maximum of 100, reported in our population at baseline. Consistent with the high symptomatic burden reported, more than a third of patients were unable to work or study. Patients’ self-reported symptoms were mainly related to the domain of orthostatic intolerance (i.e., dizziness, fainting, difficulty concentrating in the upright position, inter-scapular pain during exercise), gastrointestinal function, and pupillomotor function/visual acuity. The latter might be influenced not only by the presence of a possible rare dysautonomia [36] but also by coexisting comorbidities, namely migraine [37]. However, reduced visual acuity is more likely to be an expression of transient global cerebral hypoperfusion related to postural tachycardia.

To elicit possible gender differences in responses, we analyzed females and males separately and found no significant differences either in the demographic and clinical characteristics of the two groups or in the representation of the autonomic symptom burden at baseline. However, this result may have been influenced by the small size of the study population. 

The trend of symptoms seemed to remain substantially stable over the two years of observation. However, the patients did report a significant improvement over time, both in the overall symptomatic burden as well as in the perception of quality of life. Since the largest contribution to COMPASS 31 overall score is represented by the orthostatic intolerance domain, it is likely that the main benefit was obtained in the latter, as confirmed also by a similar trend observed in the corresponding VAS elements. Consistently, a significant improvement was observed in the pupillomotor function domain but not in the gastrointestinal function domain, whose clinical manifestations are entirely independent of the position of the body and the gravitational stress.

An interesting finding of the current study, and, to some extent, unexpected, is the improvement in autonomic symptom intensity during the follow-up, as assessed by the total COMPASS-31 score. Such perceived benefit over time could be explained by the positive effect of being taken care of by the medical staff by quarterly assessments, as well as by a new prescription or optimization of medical therapy. Indeed, the majority of patients were taking medication and most underwent changes (increase or decrease) in therapy according to the clinical judgment of the evaluating physician. Ivabradine and beta blockers were the most prescribed drugs. Non-pharmacological therapies such as physical activity as well as increased water and salt intake are the first choice in managing POTS patients, and drugs should only be added if adequate control of symptoms is not achieved [19]. Heart rate-lowering agents have proven to be effective in relieving patient symptoms; however, high quality evidence for the drug-based management of POTS is lacking [38]. It must be pointed out that our study was not designed to specifically evaluate the effect of therapy changes on symptoms. 

In the literature, only a few papers systematically describe the burden of autonomic symptoms related to POTS. Furthermore, all previous assessments were based on cross-sectional observations, and symptoms’ time-course was not evaluated.

Shaw et al. [22] conducted an online community-based survey that aimed to describe demographic and clinical features as well as the diagnostic journey of patients living with POTS. The final analysis included 4835 participants. The most common symptoms reported were lightheadedness (99%), tachycardia (97%), and presyncope (94%), all dealing with orthostatic intolerance. Headache and difficulty concentrating were also predominant (94%). Forty-four percent of patients self-reported a significant worsening of symptoms compared to diagnosis time, 29% reported a slight improvement, and 10% had reported no change. McDonald et al [28] quantified orthostatic intolerance symptoms in a population of 136 UK POTS patients using the orthostatic grading scale [39] and found that patients with POTS alone or associated with chronic fatigue syndrome had an autonomic symptom burden significantly greater than that of patients with chronic fatigue syndrome alone. Rea et al. [29] formally evaluated the autonomic symptom burden by the quantitative tool COMPASS 31 [33] in 32 patients with POTS and in a group of matched healthy controls. Additionally, they compared the assessment of POTS patients with that of a group of patients with autonomic insufficiency or neuropathy. The autonomic symptom burden in POTS patients was significantly greater than in healthy controls and had overall severity that was comparable to that of patients with other dysautonomia. Specifically, in accordance with our results, the orthostatic intolerance domain was found to be the most represented, compared both to healthy controls and to patients with other autonomic dysfunctions. The gastrointestinal function domain was found to be the second most represented in patients with POTS and predominant in patients with other dysautonomia, in the presence of the same overall COMPASS 31 score (48.05 and 50.82, respectively). Interestingly, Rea et al. found that the pupillomotor domain, in addition to the fatigue and orthostatic intolerance domains, contributed the most to clinically significant differences between POTS and healthy individuals. Based on these results, the authors therefore suggested that pupillary function evaluation should be routinely introduced in the assessment of POTS patients.

Other studies previously assessed the prognosis of POTS patients, confirming a benign course and a general long-term improvement in symptoms in the majority of patients [40,41,42]. However, they were based on retrospective data and the evaluation of symptoms was not performed in a standardized way. To our knowledge, the only prospective and standardized evaluation of the autonomic symptoms related to POTS was performed by Kimpinski et al. [43] in a cohort of 58 patients followed at the Mayo Clinic for 1 year. Similarly to what was observed in our population, orthostatic intolerance was the most severe autonomic symptom reported at baseline and had shown significant improvement at follow-up.

The results of the present study suggest that, despite the progressive global clinical improvement over the two years of observation, none of the patients who were inactive at baseline resumed their previous working activities. This may indicate the lack of specific advice by the occupational physician in pointing out alternative job tasks that are more suitable for a patient suffering from a disease characterized by orthostatic intolerance. On the other hand, patients regularly employed at baseline could maintain their job. This highlights that regular and frequent clinical assessment may promote adherence to therapy and possibly contribute to the patient’s overall wellbeing, as suggested above. 

## 5. Study Limitations

Some limitations need to be acknowledged. Firstly, our patient cohort is relatively limited in number and is characterized by a high dropout rate. This latter seemed to be related mostly to geographical difficulties in reaching our outpatient clinic. Although the baseline assessment seems to confirm the observations reported in other cross-sectional studies [22,28,33], it is possible that our prospective evaluation may have been affected by the limited number of participants. Secondly, even though an exercise program was recommended to all our patients, we could not evaluate compliance to exercise and other behavioral measures. Therefore, it is not possible to estimate the real effect of these interventions on the observed improvements in symptoms.

Finally, we chose to use the VAS scale as a semi-quantitative evaluation of autonomic symptoms, a tool not yet validated for this purpose. This tool was found to be effective in evaluating the time-course of the autonomic-related symptoms in a pure autonomic failure (PAF) patient [35], but it might be ineffective as far as POTS patients are concerned. Although the VAS results tend to confirm the main results obtained with the COMPASS 31 tool, the scale was unable to detect any statistically significant difference at the different follow-up times, except for a single item at the 2-year follow-up. This might cast doubt on its discrimination capability.

## 6. Conclusions

POTS is a clinical entity often misdiagnosed and invalidating, capable of reducing the quality of life and limiting the working ability of patients for a long time, much like other dysautonomic conditions or serious chronic pathologies. POTS prevalent symptoms are confirmed to be those of orthostatic intolerance. Awareness of the diagnosis, patient monitoring, and tailored therapies can help to improve patients’ condition and wellbeing.

## Figures and Tables

**Table 1:** Demographic and clinical features of the study population.

Demographics, Clinical Features and Hemodynamic Parameters	Patients at	Patients at	Patients at
	Baseline	1-Year Follow-Up	2-Year Follow-Up
	n = 42	n = 25	n = 12
Age (mean ± SD), y	33.9 ± 12.5	37.3 ± 12.0	42.0 ± 13.4
Female, n (%)	36 (85.7)	22 (88.0)	10 (83.3)
BMI (mean ± SD)	21.2 ± 4.0	21.4 ± 4.0	21.1 ± 4.0
Active workers, n (%)	22 (61.1) *	15 (62.5) *	7 (63.6) *
Patients with comorbidities, n (%)	22 (52.4)	13 (52.0)	5 (41.6)
Fibromyalgia	5 (11.9)	3 (12.0)	2 (16.7)
Chronic fatigue syndrome	3 (7.1)	1 (4.0)	1 (8.3)
Ehlers–Danlos syndrome	6 (14.3)	4 (16.0)	0
Chronic headache	2 (4.8)	2 (8.0)	0
Gastrointestinal disorders	4 (9.5)	2 (8.0)	1 (8.3)
Post infection	9 (21.4)	5 (20.0)	1 (8.3)
Patients on therapy, n (%) **	31 (73.8)	19 (76.0)	8 (66.7)
Beta blockers	8 (19.0)	5 (20.0)	3 (25.0)
Ivabradine	13 (31.0)	11 (44.0)	4 (33.3)
Fludrocortisone	4 (9.6)	1 (4.0)	0
Cortone acetate/prednisone	2 (4.8)	1 (4.0)	1 (8.3)
Midodrine	7 (16.7)	3 (12.0)	1 (8.3)
Benzodiazepines	4 (9.5)	1 (4.0)	1 (8.3)
Antidepressants (SSRI, SNRI, TCA)	4 (9.5)	5 (20.0)	2 (16.7)
Hemodynamic parameters			
Supine position (mean, ± SD)			
SAP (mmHg)	116.6 ± 12.6	116.5 ± 16.9	110.7 ± 15.8
DAP (mmHg)	70.2 ± 9.6	69.3 ± 11.1	69.7 ± 10.3
HR (beats/min)	80.0 ± 16.0	75.3 ± 14.7	74.4 ± 9.3
Upright position (mean, ± SD)			
SAP (mmHg)	115.8 ± 15.3	117.2 ± 19.0	118.0 ± 24.3
DAP (mmHg)	76.1 ± 12.2	78.2 ± 10.6	81.0 ± 10.9
HR (beats/min)	107.7 ± 19.3	103.0 ± 21.1	101.1 ± 15.6

BMI indicates body mass index; SSRI, selective serotonin reuptake inhibitor; SNRI, serotonin and norepinephrine reuptake inhibitor; TCA, tricyclic antidepressant; SAP, systolic arterial blood pressure; DAP, diastolic arterial blood pressure; HR, heart rate. * Work activity status was known for 36 patients out of 42 at baseline, 24 out of 25 at 1 year and 11 out of 12 at 2 years. ** Patients may assume more than 1 medication at the same time.

**Table 2:** COMPASS 31 weighted scores at baseline for the study population.

COMPASS 31 Domains (Score Range)	Baseline Weighted Score (n = 42)
Orthostatic intolerance (0–40)	27.52 ± 8.11
Vasomotor function (0–5)	2.20 ± 1.42
Secretomotor function (0–15)	5.77 ± 4.50
Gastrointestinal function (0–25)	9.37 ± 3.98
Bladder function (0–10)	2.25 ± 1.86
Pupillomotor function (0–5)	2.79 ± 1.16
Total COMPASS 31 (0–100)	49.90 ± 14.33

Results are expressed as mean ± SD.

**Table 3:** Demographic and clinical features of the baseline study population according to gender.

Demographics, Clinical Features and Hemodynamic Parameters	Females at Baseline	Males at Baseline
	n = 36	n = 6
Age (mean ± SD), y	33.8 ± 13.2	34.5 ± 7.7
BMI (mean ± SD)	20.6 ± 3.5	24.4 ± 5.2
Active workers, n (%)	20 (60.6) *	2 (66.7) *
Patients with comorbidities, n (%)	18 (50.0)	4 (66.7)
Fibromyalgia	5 (13.9)	0
Chronic fatigue syndrome	2 (5.5)	1 (16.7)
Ehlers-Danlos syndrome	6 (16.7)	0
Chronic headache	2 (5.5)	0
Gastrointestinal disorders	3 (8.3)	1 (16.7)
Post infection	7 (19.4)	2 (33.3)
Patients on therapy, n (%) **	27 (75.0)	4 (66.7)
Beta blockers	7 (19.4)	1 (16.7)
Ivabradine	11 (30.5)	2 (33.3)
Fludrocortisone	3 (8.3)	1 (16.7)
Cortone acetate/prednisone	2 (5.5)	0
Midodrine	6 (16.7)	1 (16.7)
Benzodiazepines	3 (8.3)	1 (16.7)
Antidepressants (SSRI, SNRI, TCA)	3 (8.3)	1 (16.7)
Hemodynamic parameters		
Supine position (mean, ± SD)		
SAP (mmHg)	116.5 ± 13.3	117.2 ± 7.9
DAP (mmHg)	70.5 ± 10.1	68.3 ± 5.9
HR (beats/min)	80.4 ± 16.9	78.0 ± 9.4
Upright position (mean, ± SD)		
SAP (mmHg)	116.3 ± 16.3	112.7 ± 6.2
DAP (mmHg)	75.5 ± 12.3	80.0 ± 11.8
HR (beats/min)	108.1 ± 19.9	105.3 ± 16.2

BMI indicates body mass index; SSRI, selective serotonin reuptake inhibitor; SNRI, serotonin and norepinephrine reuptake inhibitor; TCA, tricyclic antidepressant; SAP, systolic arterial blood pressure; DAP, diastolic arterial blood pressure; HR, heart rate. * Work activity status was known for 36 patients (33 females) out of 42 at baseline. ** Patients may assume more than 1 medication at the same time.

**Table 4:** COMPASS 31 weighted scores at baseline according to gender.

COMPASS 31 Domains	Baseline Weighted Score	Baseline Weighted Score
(Score Range)	Females (n = 36)	Males (n = 6)
Orthostatic intolerance (0–40)	28.00 ± 7.71	24.67 ± 10.56
Vasomotor function (0–5)	2.22 ± 1.38	2.08 ± 1.81
Secretomotor function (0–15)	5.83 ± 4.54	5.36 ± 4.65
Gastrointestinal function (0–25)	9.37 ± 3.98	9.37 ± 4.36
Bladder function (0–10)	2.13 ± 1.69	2.96 ± 2.78
Pupillomotor function (0–5)	2.84 ± 1.19	2.44 ± 1.03
Total COMPASS 31 (0–100)	50.40 ± 13.18	46.89 ± 21.31

Results are expressed as mean ± SD.

**Table 5:** COMPASS 31 autonomic symptom assessment comparison between baseline and 1-year follow-up.

COMPASS-31 Domains	Baseline (n = 25)	1-Year Follow-Up (n = 25)
Orthostatic intolerance	25.76 ± 7.49	22.88 ± 8.76
Vasomotor function	2.17 ± 1.46	2.27 ± 1.37
Secretomotor function	6.77 ± 4.32	6.00 ± 3.39
Gastrointestinal function	10.46 ± 3.44	9.57 ± 3.32
Bladder function	2.62 ± 2.00	2.31 ± 1.50
Pupillomotor function	2.85 ± 1.16	2.44 ± 1.01 *
Total COMPASS 31	50.64 ± 14.13	45.47 ± 14.54 *

* indicates *p* < 0.05 vs baseline. Results are expressed as mean ± SD.

**Table 6:** VAS autonomic symptom assessment comparison between baseline and 1-year follow-up.

VAS Domains	Baseline (n = 25)	1-Year Follow-Up (n = 25)
Orthostatic intolerance	5.90 ± 1.96	5.51 ± 1.83
Excretory function	3.23 ± 2.39	3.02 ± 1.93
Gastrointestinal function	3.17 ± 2.52	3.68 ± 2.26
Genito-urinary function	3.06 ± 2.54	3.39 ± 2.23
Reduced visual acuity	4.80 ± 2.79	4.16 ± 2.61

Results are expressed as mean ± SD.

**Table 7:** COMPASS 31 autonomic symptom assessment comparison between baseline and 1-year and 2-year follow-up.

COMPASS 31 Domains	Baseline	1-Year Follow-Up	2-Year Follow-Up
	(n = 12)	(n = 12)	(n = 12)
Orthostatic intolerance	25.67 ± 7.33	22.00 ± 6.03	21.33 ± 7.10
Vasomotor function	2.15 ± 1.40	2.08 ± 1.35	1.81 ± 1.41
Secretomotor function	6.07 ± 3.97	5.71 ± 3.91	5.71 ± 4.22
Gastrointestinal function	10.57 ± 2.92	10.42 ± 2.96	9.97 ± 3.36
Bladder function	2.87 ± 2.48	2.41 ± 1.56	2.87 ± 1.74
Pupillomotor function	2.89 ± 1.17	2.39 ± 1.20	1.94 ± 1.41 ^§^
Total COMPASS 31	50.22 ± 11.27	45.01 ± 11.75 *	43.64 ± 10.02 ^§^

* indicates *p* < 0.05 1 year vs. baseline; ^§^ indicates *p* < 0.05 2 years vs. baseline. Results are expressed as mean ± SD.

**Table 8:** VAS autonomic symptom assessment comparison between baseline and 1-year and 2-year follow-up.

VAS Domains	Baseline	1-Year Follow-Up	2-Year Follow-Up
	(n = 12)	(n = 12)	(n = 12)
Orthostatic intolerance	6.23 ± 1.83	5.63 ± 1.66	5.19 ± 1.79
Excretory function	3.69 ± 2.50	2.90 ± 2.18	2.98 ± 2.22
Gastrointestinal function	3.80 ± 2.75	3.76 ± 2.25	3.87 ± 2.37
Genito-urinary function	3.06 ± 2.78	3.59 ± 2.48	3.03 ± 2.33
Visual acuity	5.63 ± 2.97	4.64 ± 3.14	4.09 ± 2.91

* indicates *p* < 0.05 vs. baseline. Results are expressed as mean ± SD.
